# Crystal structure of 9-amino­acridinium chloride *N*,*N*-di­methyl­formamide monosolvate

**DOI:** 10.1107/S2056989021011816

**Published:** 2021-11-16

**Authors:** Igor O. Fritsky, Valerii Y. Sirenko, Sergiu Shova, Olesia I. Kucheriv, Il’ya A. Gural’skiy

**Affiliations:** a Department of Chemistry, Taras Shevchenko National University of Kyiv, Volodymyrska St. 64, Kyiv 01601, Ukraine; b Department of Inorganic Polymers, "Petru Poni" Institute of Macromolecular Chemistry, Romanian Academy of Science, Aleea Grigore Ghica Voda 41-A, Iasi, 700487, Romania

**Keywords:** crystal structure, 9-amino­acridinium, hydrogen bonds

## Abstract

9-Amino­acridinium chloride *N*,*N*-di­methyl­formamide monosolvate, C_13_H_11_N_2_^+^Cl^−^·C_3_H_7_NO, crystallizes in the monoclinic space group *P*2_1_/*c.* The salt was crystallized from *N*,*N*-di­methyl­formamide. The asymmetric unit consists of two C_13_H_11_N_2_^+^Cl^−^ formula units. The 9-amino­acridinium (9-AA) mol­ecules are protonated with the proton on the N atom of the central ring. This N atom is connected to an *N*,*N*-di­methyl­formamide mol­ecule by a hydrogen bond. The H atoms of the amino groups create short contacts with two chloride ions. The 9-AA cations in adjacent layers are oriented in an anti­parallel manner. The mol­ecules are linked *via* a network of multidirectional π–π inter­actions between the 9-AA rings, and the whole lattice is additionally stabilized by electrostatic inter­actions between ions.

## Abstract 2

9-Amino­acridinium chloride *N*,*N*-di­methyl­formamide monosolvate was found to crystallize in the monoclinic space group *P*2_1_/*c*. The crystal structure of this compound is stabilized by N—H⋯O and N—H⋯Cl hydrogen bonds, as well as π–π stacking.

## Chemical context

Amino­acridine (AA) derivatives exhibit anti­bacterial (Ciric *et al.*, 2011 ▸), anti­cancer (Hassan *et al.*, 2011 ▸), anti­viral (Kaur & Singh, 2011 ▸) and anti­prion effects (Villa *et al.*, 2011 ▸), as well as other therapeutic properties (Muregi & Ishih, 2010 ▸). The synthesis of these compounds and analysis of their inter­actions is very useful in view of their importance in a wide range of different biological systems (Coupar *et al.*, 1997 ▸). Besides, numerous acridine-based derivatives are important for their chemiluminogenic ability and their use as chemiluminescent indicators in immunoassays, nucleic acid diagnostics and quan­ti­tative assays of biomolecules, such as anti­gens, anti­bodies, hormones and enzymes, as well as DNA–RNA structural analyses (Dodeigne, 2000 ▸; Becker *et al.*, 1999 ▸). Additionally, photochemical reactions for these compounds in different media have been reported (Machulek *et al.*, 2003 ▸). AA derivatives are promising analytical agents, since they exhibit relatively high quantum yields of light emission and stability (Adamczyk *et al.*, 1999 ▸; Dodeigne, 2000 ▸; Renotte *et al.*, 2000 ▸; Smith *et al.*, 2009 ▸).

9-AA is a fluorescent dye of the family of nitro­gen heterocyclic bases. 9-AA has been proposed as a specific fluorescent probe capable of binding the active center of guanidinobenzoatases (GB) (Murza *et al.*, 2000 ▸). Inter­estingly, cellulose nanocomposites based on [Fe(hptrz)_3_](OTs)_2_ nanoparticles were effectively doped with 9-AA, resulting in a thermochromic and thermofluorescent material (Nagy *et al.*, 2014 ▸). Previous crystallographic studies of some analogues of 9-AA have revealed that while in some members the acridine ring system is nearly planar (Carrell, 1972 ▸), in others it is clamped (Zacharias & Glusker, 1974 ▸; Berman & Glusker, 1972 ▸; Glusker *et al.*, 1973 ▸) with angles of 7–13° between the two outer rings. This publication reports the crystal structure of 9-amino­acridinium chloride *N*,*N*-di­methyl­formamide solvate (1:1). 
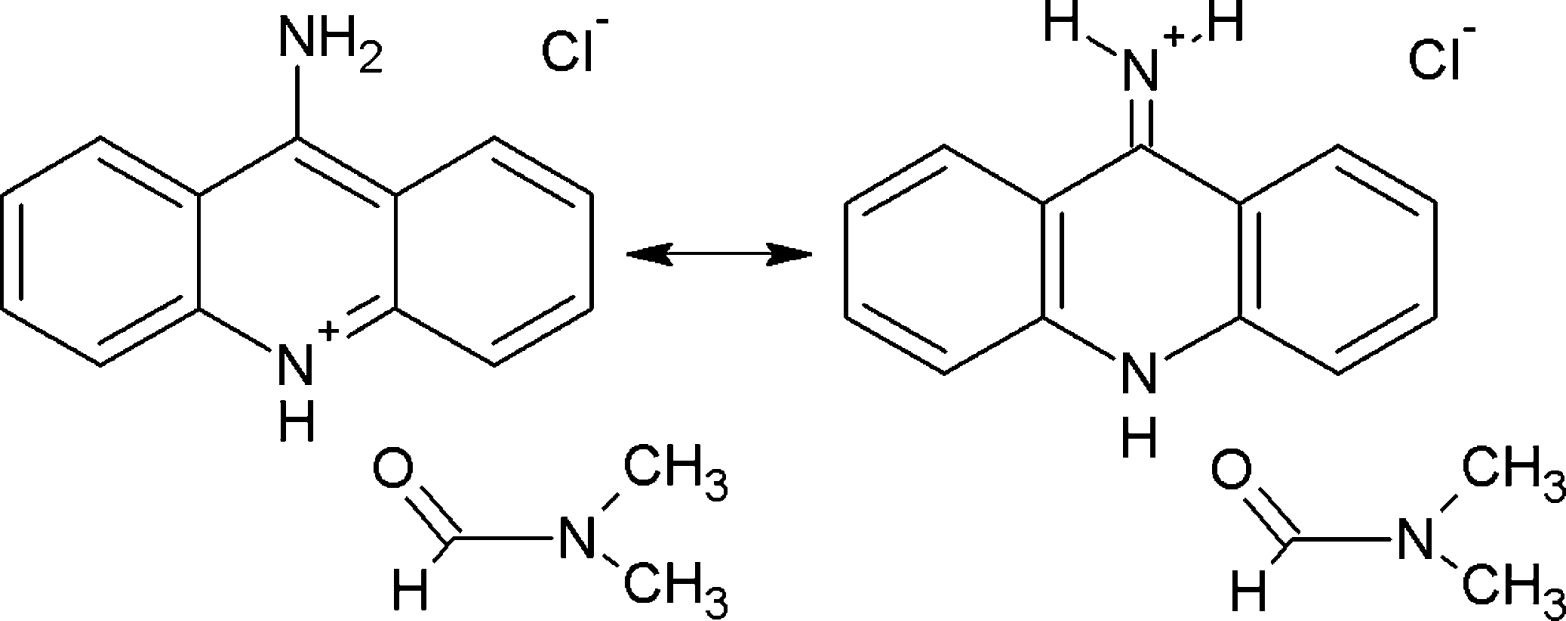


## Structural commentary

**Figure 1:**
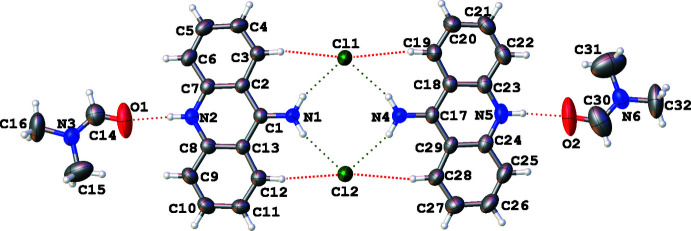
The mol­ecular structure of the title compound, showing the atom-labelling scheme and displacement ellipsoids drawn at the 50% probability level. H atoms are shown as small spheres of arbitrary radii. Hydrogen bonds are represented by dashed lines. Two amine groups and two chloride ions form a supra­molecular 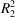 (8) synthon.

The title compound crystallizes in the monoclinic *P*2_1_/*c* space group, with two 9-AA^+^Cl^−^ formula units in the asymmetric unit. As shown in Fig. 1 ▸, the mol­ecules are monoionized with the one proton residing on the N atom, N2 or N5, of the central ring.

The amino groups for two 9-amino­acridine mol­ecules do not readily add a proton. The state of ionization is confirmed by both the H-atom positions (located from the difference map) and by the hydrogen bonding as shown in Table 1 ▸. The C—NH_2_ bonds C1—N1 and C17—N4 are 1.310 (5) and 1.313 (5) Å, respectively. These bond lengths are characteristic for a C=N double bond that can originate from tautomerism of the cation, as shown on the scheme.

The acridine moieties are nearly planar in the crystalline phase with atoms N2, C1, N1 and N5, C17 and N4 arranged almost linearly (N2⋯C1—N1 = 176° and N5⋯C17—N4 = 180°). The dihedral angle between the two outer fused rings is 3.39 (14)° for the mol­ecule containing N2, while the corresponding angle in the mol­ecule containing N5 is 1.18 (15)°. The second value is comparable with that found for acridine (1.2°; Phillips, 1956 ▸; Phillips *et al.*, 1960 ▸). The 9-AA mol­ecules are almost planar and each of three fused rings taken individually is planar within experimental error.

## Supra­molecular features

**Figure 2:**
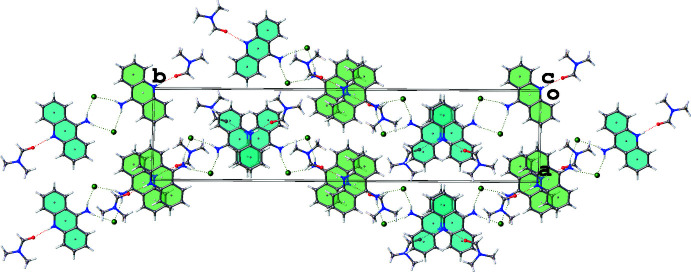
Crystal packing viewed along the *c* axis. The N—H⋯Cl and N—H⋯O inter­actions are represented by green and red dashed lines, respectively. The *A* and *B* acridine mol­ecules are coloured green and blue, respectively.

**Figure 3:**
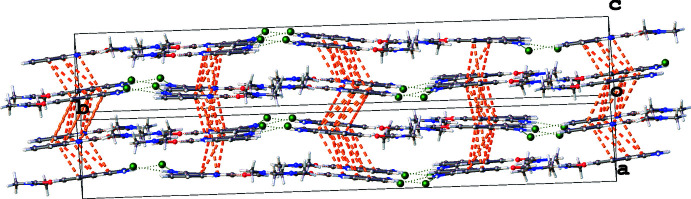
Layers of 9-AA. π–π stacking inter­actions between the 9-amino­acridinium rings of different layers are shown by orange dashed lines.

The packing of the mol­ecules in the crystal is illustrated in Fig. 2 ▸. The crystal structure features N—H⋯O and N—H⋯Cl hydrogen bonds (Table 1 ▸) as well as π–π stacking inter­actions. The 9-AA mol­ecules form layers (Fig. 3 ▸ *)*, which stack perpendicularly to the *c* axis. There are two types of 9-AA fused rings in the crystal structure, which results in the propagation of layers in a zigzag manner along *b-*axis direction (Fig. 2 ▸ *)*.

The structure is characterized by the presence of several different kinds of weak inter­actions, which create a three-dimensional supra­molecular network. The atoms H2 and H5*A*, attached to N2 and N5, form hydrogen bonds to *N*,*N*-di­methyl­formamide atoms, O1 and O2, with *d*(N⋯O) = 2.723 (5)–2.740 (5) Å, N—H⋯O = 175-176°. The chloride ions are linked *via* N—H⋯Cl hydrogen bonds [*d*(N⋯Cl) = 3.209 (3)–3.246 (3) Å, N-H⋯Cl = 160–163°], forming di­mers (Fig. 1 ▸). In these di­mers, the amino groups of the two 9-AA cations and the two halide anions participate in the hydrogen bonding, generating a centrosymmetric 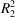 (8) supra­molecular synthon (Etter, 1990 ▸; Etter *et al.*, 1990 ▸; Aakeröy, 1997 ▸). The di­mers are also stabilized by C—H⋯Cl hydrogen bonds between C atoms in positions 1 and 8 in the 9-AA skeleton and the halide ions [*d*(C⋯Cl) = 3.608 (5)–3.688 (4) Å and C—H⋯Cl = 163-172°] (Fig. 2 ▸), as is also observed in other 9-AA salts (Sikorski & Trzybiński, 2011*a* ▸,*b* ▸; 2013 ▸).

Adjacent acridine skeletons are linked *via* π–π stacking inter­actions in an *AB* arrangement (Fig. 3 ▸). All of the aromatic rings of the *A* mol­ecules participate in π–π inter­actions, propagating in zigzag manner along the *c-*axis direction with centroid–centroid distances ranging from 3.9786 (3) to 4.2236 (3) Å. On the other hand, only the two aromatic rings of the acridine *B* mol­ecules participate in π–π inter­actions, with adjacent acridine skeletons rotated in-plane with respect to one another. The centroid–centroid distances vary from 3.6514 (3) to 4.7445 (5) Å.

## Database survey

A search of the Cambridge Structure Database (CSD version 5.42, last update February 2021; Groom *et al.*, 2016 ▸) revealed that the current structure has never been published before. 101 structures containing 9-AA cations and chloride anions were found. These include 9-amino­acridine hydro­chloride monohydrate (refcode: AMACRD; Talacki *et al.*, 1974 ▸), which consists of a monoionized 9-amino­acride mol­ecule with the proton on the N atom of the central ring, one water mol­ecule, which is hydrogen bonded to another water mol­ecule, and two chloride ions, which are hydrogen bonded to the amino group of the 9-AA cation. 9-Amino­acridinium 3-chloro­benzoate (AQAGEF; Sikorski & Trzybiński, 2011*b* ▸) crystallizes in the monoclinic *P*2_1_/c space group with an 9-AA cation and a 3-chloro­benzoate anion in the asymmetric unit and the crystal structure features N—H⋯O and C—H⋯O hydrogen bonds and π–π stacking inter­actions. Inversely oriented cations and anions form a tetra­mer; these ions are linked *via* N(amino)—H⋯O (carb­oxy) hydrogen bonds, forming a ring motif. 9-Amino­acridinium 3-chloro­benzoate (AQAGIJ; (Sikorski & Trzybiński, 2011*b* ▸) forms triclinic crystals (*P* 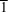 space group) with an 9-AA cation, a 4-chloro­benzoate anion and a water mol­ecule in the asymmetric unit. The crystal structure features N—H⋯O and O—H⋯O hydrogen bonds and π–π inter­actions. Analysis of the hydrogen bonds in the structure of this compounds shows that the ions form tetra­mers and produce an 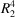 (16) hydrogen-bond ring motif. 9-Amino­acridinium 3-hy­droxy­benzoate (AQAGOP; Sikorski & Trzybiński, 2011*b* ▸) also crystallizes in the triclinic *P* 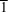 space group, the asymmetric unit consisting of two 9-AA cations, 3-hy­droxy­benzoate and chlorate anions as well as two water mol­ecules. This structure is the first of all the known 9-amino­acridinium salts where mixed salts were obtained (Allen, 2002 ▸). The average deviations from planarity of the acridine skeleton are 0.015 (2) and 0.027 (2) Å, and the angle between the mean planes of the right- and left-hand halves of the acridine skeleton is 1.5 and 3.7° in cations *A* and *B*, respectively. Analysis of the hydrogen bonds in this compound shows that the ions do not form tetra­mers, but produce two nearly perpendicularly aligned kinds of hydrogen-bonded chain motif. 9-Amino­acridinium chloride methanol solvate (SIDHAQ; Trzybiński & Sikorski, 2013 ▸) again forms triclinic crystals (*P* 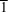 space group). The amino group of the 9-amino­acridinium cation inter­acts with the chloride anion *via* an N—H⋯Cl hydrogen bond and the methanol mol­ecule *via* an N—H⋯O hydrogen bond, generating a centrosymmetric 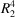 (16) supra­molecular synthon. The methanol mol­ecule inter­acts with the halide ion; the resulting supra­molecular synthon 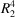 (12) is not planar but assumes a chair shape. This hydrogen-bonded ring motif is stabilized by the N—H⋯Cl hydrogen bond between the acridinium skeleton and the halide ion.

## Synthesis and crystallization

9-Amino­acridinium hydro­chloride (0.0624 g, 2.71×10 ^−4^ mol) was dissolved in *N*,*N*-di­methyl­formamide (4 ml) under heating at 418 K until the 9-AA·HCl had fully dissolved. The solution was left to cool to 280 K. Single crystals were obtained after 2 days.

## Refinement

Crystal data, data collection and structure refinement details are summarized in Table 2 ▸. All H atoms were placed geom­etrically and refined as riding, with C—H = 0.93 Å and *U*_iso_(H) = 1.2*U*_eq_(C) for aromatic hydrogens and the C—H group and C—H = 0.96 Å and *U*_iso_(H) = 1.5*U*_eq_(C) for the CH_3_ group. A rotating model was used for the methyl group.

## Supplementary Material

Crystal structure: contains datablock(s) I. DOI: 10.1107/S2056989021011816/dx2038sup1.cif

Structure factors: contains datablock(s) I. DOI: 10.1107/S2056989021011816/dx2038Isup2.hkl

Supporting information file. DOI: 10.1107/S2056989021011816/dx2038Isup3.cml

CCDC reference: 2120699

Additional supporting information:  crystallographic information; 3D view; checkCIF report

## Figures and Tables

**Table 1:** Hydrogen-bond geometry (Å, °)

*D*—H⋯*A*	*D*—H	H⋯*A*	*D*⋯*A*	*D*—H⋯*A*
N2—H2⋯O1	0.86	1.86	2.723 (5)	176
N4—H4*A*⋯Cl2	0.86	2.40	3.225 (3)	160
N4—H4*B*⋯Cl1	0.86	2.38	3.211 (4)	163
N1—H1*A*⋯Cl1	0.86	2.39	3.209 (3)	160
N1—H1*B*⋯Cl2	0.86	2.42	3.246 (3)	162
N5—H5*A*⋯O2	0.86	1.88	2.740 (5)	175

**Table 2:** Experimental details

Crystal data	
Chemical formula	C_13_H_11_N_2_^+^·Cl^−^·C_3_H_7_NO
*M*_r_	303.78
Crystal system, space group	Monoclinic, *P*2_1_/*c*
Temperature (K)	293
*a*, *b*, *c* (Å)	10.5819 (7), 42.705 (2), 7.2531 (6)
β (°)	108.800 (8)
*V* (Å^3^)	3102.8 (4)
*Z*	8
Radiation type	Mo *K*α
μ (mm^−1^)	0.25
Crystal size (mm)	0.3 × 0.2 × 0.15

Data collection	
Diffractometer	Xcalibur, Eos
Absorption correction	Multi-scan (*CrysAlis PRO*; Rigaku OD, 2019 ▸)
*T*_min_, *T*_max_	0.955, 1.000
No. of measured, independent and observed [*I* > 2σ(*I*)] reflections	12374, 5491, 3496
*R*_int_	0.040
(sin θ/λ)_max_ (Å^−1^)	0.595

Refinement	
*R*[*F* ^2^ > 2σ(*F* ^2^)], *wR*(*F* ^2^), *S*	0.085, 0.199, 1.10
No. of reflections	5491
No. of parameters	383
H-atom treatment	H-atom parameters constrained
Δρ_max_, Δρ_min_ (e Å^−3^)	0.58, −0.27

Computer programs: *CrysAlis PRO* (Rigaku OD, 2019 ▸), *SHELXT* (Sheldrick, 2015*a* ▸), *SHELXL2018/3* (Sheldrick, 2015*b* ▸) and *OLEX2* (Dolomanov *et al.*, 2009 ▸ ▸).

## supplementary crystallographic information

### Crystal data

**Table:**

C_13_H_11_N_2_^+^·Cl^−^·C_3_H_7_NO	*F*(000) = 1280
*M**_r_* = 303.78	*D*_x_ = 1.301 Mg m^−^^3^
Monoclinic, *P*2_1_/*c*	Mo *K*α radiation, λ = 0.71073 Å
*a* = 10.5819 (7) Å	Cell parameters from 3798 reflections
*b* = 42.705 (2) Å	θ = 2.1–26.7°
*c* = 7.2531 (6) Å	µ = 0.25 mm^−^^1^
β = 108.800 (8)°	*T* = 293 K
*V* = 3102.8 (4) Å^3^	Block, clear intense yellow
*Z* = 8	0.3 × 0.2 × 0.15 mm

### Data collection

**Table:**

Xcalibur, Eos diffractometer	3496 reflections with *I* > 2σ(*I*)
Detector resolution: 8.0797 pixels mm^-1^	*R*_int_ = 0.040
ω scans	θ_max_ = 25.0°, θ_min_ = 1.9°
Absorption correction: multi-scan (CrysAlisPro; Rigaku OD, 2019)	*h* = −8→12
*T*_min_ = 0.955, *T*_max_ = 1.000	*k* = −34→50
12374 measured reflections	*l* = −8→8
5491 independent reflections	

### Refinement

**Table:**

Refinement on *F*^2^	0 restraints
Least-squares matrix: full	Hydrogen site location: inferred from neighbouring sites
*R*[*F*^2^ > 2σ(*F*^2^)] = 0.085	H-atom parameters constrained
*wR*(*F*^2^) = 0.199	*w* = 1/[σ^2^(*F*_o_^2^) + (0.0638*P*)^2^ + 2.1432*P*] where *P* = (*F*_o_^2^ + 2*F*_c_^2^)/3
*S* = 1.10	(Δ/σ)_max_ = 0.001
5491 reflections	Δρ_max_ = 0.58 e Å^−^^3^
383 parameters	Δρ_min_ = −0.27 e Å^−^^3^

### Special details

**Table:**

Geometry. All esds (except the esd in the dihedral angle between two l.s. planes) are estimated using the full covariance matrix. The cell esds are taken into account individually in the estimation of esds in distances, angles and torsion angles; correlations between esds in cell parameters are only used when they are defined by crystal symmetry. An approximate (isotropic) treatment of cell esds is used for estimating esds involving l.s. planes.

### Fractional atomic coordinates and isotropic or equivalent isotropic displacement parameters (Å^2^)

**Table:**

	*x*	*y*	*z*	*U*_iso_*/*U*_eq_	
Cl2	0.52995 (11)	0.60004 (2)	0.42541 (19)	0.0646 (4)	
Cl1	0.92064 (11)	0.65087 (2)	0.79353 (19)	0.0652 (4)	
N2	0.4780 (3)	0.75521 (8)	0.2516 (5)	0.0473 (9)	
H2	0.439961	0.772953	0.214055	0.057*	
N4	0.8349 (3)	0.58046 (8)	0.6530 (5)	0.0558 (10)	
H4A	0.753411	0.581081	0.577485	0.067*	
H4B	0.875157	0.597465	0.702537	0.067*	
N1	0.6495 (3)	0.67060 (8)	0.4611 (5)	0.0546 (10)	
H1A	0.729108	0.669943	0.542722	0.066*	
H1B	0.604150	0.653625	0.427738	0.066*	
N5	1.0324 (4)	0.49648 (8)	0.7759 (5)	0.0572 (10)	
H5A	1.074306	0.478926	0.801115	0.069*	
N3	0.1963 (4)	0.84737 (8)	−0.0005 (6)	0.0591 (10)	
N6	1.3057 (4)	0.40331 (10)	0.9714 (6)	0.0646 (11)	
C2	0.6704 (4)	0.72613 (9)	0.4405 (6)	0.0407 (10)	
C7	0.6074 (4)	0.75488 (9)	0.3723 (6)	0.0419 (10)	
C29	0.8324 (4)	0.52493 (9)	0.6113 (6)	0.0441 (10)	
C1	0.5973 (4)	0.69747 (9)	0.3876 (6)	0.0411 (10)	
C13	0.4638 (4)	0.69898 (9)	0.2499 (6)	0.0415 (10)	
C17	0.8978 (4)	0.55353 (9)	0.6925 (6)	0.0433 (10)	
C8	0.4070 (4)	0.72836 (9)	0.1884 (6)	0.0427 (10)	
C18	1.0344 (4)	0.55202 (10)	0.8188 (6)	0.0469 (10)	
C12	0.3863 (4)	0.67215 (10)	0.1781 (6)	0.0502 (11)	
H12	0.421945	0.652389	0.216721	0.060*	
C3	0.8063 (4)	0.72726 (10)	0.5627 (6)	0.0478 (11)	
H3	0.851443	0.708777	0.610598	0.057*	
C23	1.0976 (4)	0.52306 (10)	0.8578 (6)	0.0486 (11)	
C11	0.2594 (4)	0.67488 (11)	0.0522 (6)	0.0575 (12)	
H11	0.209574	0.656968	0.004566	0.069*	
C6	0.6752 (5)	0.78324 (10)	0.4249 (6)	0.0533 (12)	
H6	0.631891	0.802055	0.380356	0.064*	
C24	0.9034 (5)	0.49659 (10)	0.6557 (6)	0.0496 (11)	
O1	0.3482 (5)	0.81000 (10)	0.1214 (7)	0.1213 (17)	
C19	1.1073 (4)	0.57874 (11)	0.9076 (7)	0.0578 (12)	
H19	1.067290	0.598375	0.884741	0.069*	
C28	0.6990 (4)	0.52341 (10)	0.4876 (7)	0.0593 (12)	
H28	0.649844	0.541808	0.454043	0.071*	
C9	0.2745 (4)	0.73091 (11)	0.0596 (7)	0.0575 (12)	
H9	0.236582	0.750446	0.020291	0.069*	
C4	0.8703 (4)	0.75517 (10)	0.6096 (6)	0.0559 (12)	
H4	0.959115	0.755529	0.688614	0.067*	
C5	0.8052 (5)	0.78322 (11)	0.5416 (6)	0.0595 (13)	
H5	0.850566	0.802089	0.575935	0.071*	
C22	1.2312 (5)	0.52056 (12)	0.9817 (7)	0.0634 (13)	
H22	1.273053	0.501138	1.006910	0.076*	
C10	0.2037 (5)	0.70426 (11)	−0.0055 (7)	0.0625 (13)	
H10	0.116652	0.705698	−0.089990	0.075*	
C25	0.8413 (5)	0.46818 (11)	0.5788 (7)	0.0640 (13)	
H25	0.889114	0.449551	0.608286	0.077*	
C21	1.2971 (5)	0.54672 (12)	1.0626 (7)	0.0657 (14)	
H21	1.384991	0.545175	1.144215	0.079*	
C20	1.2366 (5)	0.57594 (12)	1.0269 (7)	0.0649 (13)	
H20	1.284081	0.593680	1.084266	0.078*	
C14	0.3158 (6)	0.83691 (14)	0.0906 (8)	0.0750 (15)	
H14	0.382748	0.851803	0.136486	0.090*	
C26	0.7123 (5)	0.46777 (11)	0.4620 (7)	0.0709 (15)	
H26	0.671583	0.448903	0.412320	0.085*	
C27	0.6406 (5)	0.49580 (12)	0.4165 (7)	0.0689 (14)	
H27	0.552007	0.495458	0.336611	0.083*	
O2	1.1518 (6)	0.43877 (11)	0.8412 (8)	0.149 (2)	
C16	0.1695 (6)	0.88039 (13)	−0.0371 (11)	0.115 (2)	
H16A	0.248518	0.892193	0.028244	0.172*	
H16B	0.098683	0.886614	0.010836	0.172*	
H16C	0.143641	0.884297	−0.174604	0.172*	
C32	1.3588 (7)	0.37299 (14)	1.0227 (10)	0.121 (3)	
H32A	1.371578	0.369283	1.158012	0.181*	
H32B	1.442925	0.371355	0.999575	0.181*	
H32C	1.297962	0.357724	0.945223	0.181*	
C30	1.1894 (8)	0.4107 (2)	0.8729 (11)	0.127 (3)	
H30	1.127945	0.394876	0.820824	0.152*	
C15	0.0865 (6)	0.82581 (15)	−0.0790 (11)	0.120 (3)	
H15A	0.056623	0.826913	−0.218681	0.180*	
H15B	0.014377	0.831333	−0.032063	0.180*	
H15C	0.115624	0.804886	−0.038441	0.180*	
C31	1.3999 (9)	0.42835 (19)	1.0510 (12)	0.158 (3)	
H31A	1.449984	0.423680	1.183929	0.237*	
H31B	1.352309	0.447662	1.045383	0.237*	
H31C	1.459666	0.430308	0.976317	0.237*	

### Atomic displacement parameters (Å^2^)

**Table:**

	*U*^11^	*U*^22^	*U*^33^	*U*^12^	*U*^13^	*U*^23^
Cl2	0.0509 (7)	0.0407 (6)	0.0845 (9)	0.0001 (5)	−0.0026 (6)	0.0013 (6)
Cl1	0.0487 (7)	0.0370 (6)	0.0911 (9)	0.0017 (5)	−0.0038 (6)	−0.0027 (6)
N2	0.049 (2)	0.039 (2)	0.050 (2)	0.0050 (16)	0.0093 (17)	0.0074 (17)
N4	0.043 (2)	0.039 (2)	0.075 (3)	0.0002 (16)	0.0048 (18)	−0.0053 (19)
N1	0.044 (2)	0.0325 (19)	0.073 (3)	−0.0036 (15)	−0.0020 (18)	0.0044 (18)
N5	0.065 (3)	0.042 (2)	0.061 (3)	0.0161 (19)	0.017 (2)	0.0092 (19)
N3	0.058 (2)	0.044 (2)	0.074 (3)	0.0020 (19)	0.020 (2)	0.004 (2)
N6	0.054 (2)	0.064 (3)	0.077 (3)	0.012 (2)	0.023 (2)	0.014 (2)
C2	0.046 (2)	0.035 (2)	0.040 (2)	−0.0028 (18)	0.0128 (19)	0.0019 (19)
C7	0.049 (2)	0.038 (2)	0.038 (2)	−0.0003 (19)	0.014 (2)	0.0001 (19)
C29	0.054 (3)	0.033 (2)	0.048 (3)	0.0019 (19)	0.021 (2)	0.000 (2)
C1	0.042 (2)	0.039 (2)	0.041 (2)	0.0038 (18)	0.0114 (18)	−0.0004 (19)
C13	0.042 (2)	0.037 (2)	0.043 (2)	0.0002 (18)	0.0107 (18)	0.0056 (19)
C17	0.048 (2)	0.038 (2)	0.045 (2)	0.0074 (19)	0.0157 (19)	0.000 (2)
C8	0.043 (2)	0.046 (3)	0.038 (2)	−0.0017 (19)	0.0116 (18)	0.005 (2)
C18	0.048 (2)	0.045 (3)	0.048 (3)	0.007 (2)	0.014 (2)	0.002 (2)
C12	0.049 (3)	0.045 (3)	0.052 (3)	0.000 (2)	0.010 (2)	0.003 (2)
C3	0.045 (2)	0.043 (3)	0.048 (3)	0.0000 (19)	0.005 (2)	0.004 (2)
C23	0.055 (3)	0.046 (3)	0.045 (3)	0.008 (2)	0.016 (2)	0.004 (2)
C11	0.046 (3)	0.055 (3)	0.063 (3)	−0.008 (2)	0.004 (2)	−0.001 (2)
C6	0.070 (3)	0.036 (2)	0.049 (3)	−0.005 (2)	0.012 (2)	0.004 (2)
C24	0.065 (3)	0.040 (3)	0.048 (3)	0.004 (2)	0.023 (2)	0.002 (2)
O1	0.159 (4)	0.091 (3)	0.131 (4)	0.074 (3)	0.070 (3)	0.058 (3)
C19	0.052 (3)	0.046 (3)	0.069 (3)	0.007 (2)	0.011 (2)	−0.004 (2)
C28	0.059 (3)	0.041 (3)	0.071 (3)	0.000 (2)	0.011 (2)	−0.009 (2)
C9	0.045 (3)	0.056 (3)	0.062 (3)	0.005 (2)	0.004 (2)	0.010 (2)
C4	0.055 (3)	0.050 (3)	0.052 (3)	−0.013 (2)	0.002 (2)	0.005 (2)
C5	0.075 (3)	0.045 (3)	0.051 (3)	−0.016 (2)	0.010 (2)	0.000 (2)
C22	0.058 (3)	0.066 (3)	0.063 (3)	0.022 (3)	0.015 (2)	0.005 (3)
C10	0.048 (3)	0.063 (3)	0.063 (3)	0.001 (2)	−0.001 (2)	0.012 (3)
C25	0.088 (4)	0.038 (3)	0.066 (3)	0.005 (2)	0.024 (3)	−0.002 (2)
C21	0.050 (3)	0.074 (4)	0.064 (3)	0.013 (3)	0.006 (2)	0.008 (3)
C20	0.054 (3)	0.059 (3)	0.070 (3)	0.001 (2)	0.004 (2)	−0.004 (3)
C14	0.077 (4)	0.073 (4)	0.074 (4)	0.009 (3)	0.023 (3)	0.007 (3)
C26	0.092 (4)	0.043 (3)	0.073 (4)	−0.007 (3)	0.019 (3)	−0.013 (3)
C27	0.066 (3)	0.060 (3)	0.074 (3)	−0.004 (3)	0.012 (3)	−0.014 (3)
O2	0.183 (5)	0.100 (4)	0.181 (5)	0.093 (4)	0.082 (4)	0.065 (4)
C16	0.119 (5)	0.063 (4)	0.169 (7)	0.036 (4)	0.056 (5)	0.030 (4)
C32	0.160 (7)	0.084 (5)	0.126 (6)	0.069 (4)	0.060 (5)	0.032 (4)
C30	0.111 (6)	0.147 (7)	0.122 (6)	0.048 (5)	0.039 (5)	0.044 (5)
C15	0.102 (5)	0.108 (5)	0.157 (7)	−0.042 (4)	0.051 (5)	−0.040 (5)
C31	0.180 (8)	0.153 (8)	0.142 (7)	−0.073 (7)	0.053 (6)	−0.008 (6)

### Geometric parameters (Å, º)

**Table:**

N2—H2	0.8600	C6—H6	0.9300
N2—C7	1.367 (5)	C6—C5	1.363 (6)
N2—C8	1.367 (5)	C24—C25	1.407 (6)
N4—H4A	0.8600	O1—C14	1.199 (6)
N4—H4B	0.8600	C19—H19	0.9300
N4—C17	1.313 (5)	C19—C20	1.368 (6)
N1—H1A	0.8600	C28—H28	0.9300
N1—H1B	0.8600	C28—C27	1.354 (6)
N1—C1	1.310 (5)	C9—H9	0.9300
N5—H5A	0.8600	C9—C10	1.361 (6)
N5—C23	1.361 (5)	C4—H4	0.9300
N5—C24	1.362 (5)	C4—C5	1.391 (6)
N3—C14	1.303 (6)	C5—H5	0.9300
N3—C16	1.446 (6)	C22—H22	0.9300
N3—C15	1.448 (6)	C22—C21	1.347 (6)
N6—C32	1.413 (6)	C10—H10	0.9300
N6—C30	1.248 (7)	C25—H25	0.9300
N6—C31	1.449 (7)	C25—C26	1.355 (7)
C2—C7	1.409 (5)	C21—H21	0.9300
C2—C1	1.433 (5)	C21—C20	1.388 (6)
C2—C3	1.426 (5)	C20—H20	0.9300
C7—C6	1.397 (5)	C14—H14	0.9300
C29—C17	1.434 (5)	C26—H26	0.9300
C29—C24	1.406 (5)	C26—C27	1.399 (6)
C29—C28	1.410 (6)	C27—H27	0.9300
C1—C13	1.445 (5)	O2—C30	1.260 (8)
C13—C8	1.401 (5)	C16—H16A	0.9600
C13—C12	1.408 (5)	C16—H16B	0.9600
C17—C18	1.441 (5)	C16—H16C	0.9600
C8—C9	1.416 (5)	C32—H32A	0.9600
C18—C23	1.391 (5)	C32—H32B	0.9600
C18—C19	1.412 (6)	C32—H32C	0.9600
C12—H12	0.9300	C30—H30	0.9300
C12—C11	1.364 (6)	C15—H15A	0.9600
C3—H3	0.9300	C15—H15B	0.9600
C3—C4	1.359 (5)	C15—H15C	0.9600
C23—C22	1.414 (6)	C31—H31A	0.9600
C11—H11	0.9300	C31—H31B	0.9600
C11—C10	1.391 (6)	C31—H31C	0.9600
			
C7—N2—H2	118.8	C29—C28—H28	119.2
C7—N2—C8	122.3 (3)	C27—C28—C29	121.5 (4)
C8—N2—H2	118.8	C27—C28—H28	119.2
H4A—N4—H4B	120.0	C8—C9—H9	120.6
C17—N4—H4A	120.0	C10—C9—C8	118.8 (4)
C17—N4—H4B	120.0	C10—C9—H9	120.6
H1A—N1—H1B	120.0	C3—C4—H4	119.4
C1—N1—H1A	120.0	C3—C4—C5	121.1 (4)
C1—N1—H1B	120.0	C5—C4—H4	119.4
C23—N5—H5A	118.7	C6—C5—C4	120.4 (4)
C23—N5—C24	122.6 (4)	C6—C5—H5	119.8
C24—N5—H5A	118.7	C4—C5—H5	119.8
C14—N3—C16	121.9 (5)	C23—C22—H22	120.5
C14—N3—C15	120.4 (5)	C21—C22—C23	119.0 (4)
C16—N3—C15	117.6 (5)	C21—C22—H22	120.5
C32—N6—C31	114.0 (6)	C11—C10—H10	119.4
C30—N6—C32	128.2 (6)	C9—C10—C11	121.2 (4)
C30—N6—C31	117.7 (6)	C9—C10—H10	119.4
C7—C2—C1	119.8 (3)	C24—C25—H25	119.8
C7—C2—C3	117.2 (3)	C26—C25—C24	120.5 (4)
C3—C2—C1	123.0 (4)	C26—C25—H25	119.8
N2—C7—C2	119.8 (4)	C22—C21—H21	119.3
N2—C7—C6	119.1 (4)	C22—C21—C20	121.4 (4)
C6—C7—C2	121.1 (4)	C20—C21—H21	119.3
C24—C29—C17	119.2 (4)	C19—C20—C21	120.2 (5)
C24—C29—C28	117.3 (4)	C19—C20—H20	119.9
C28—C29—C17	123.6 (4)	C21—C20—H20	119.9
N1—C1—C2	121.2 (4)	N3—C14—H14	116.7
N1—C1—C13	120.7 (4)	O1—C14—N3	126.6 (6)
C2—C1—C13	118.0 (3)	O1—C14—H14	116.7
C8—C13—C1	118.9 (4)	C25—C26—H26	120.1
C8—C13—C12	118.2 (4)	C25—C26—C27	119.8 (5)
C12—C13—C1	122.9 (4)	C27—C26—H26	120.1
N4—C17—C29	120.8 (4)	C28—C27—C26	120.6 (5)
N4—C17—C18	120.8 (4)	C28—C27—H27	119.7
C29—C17—C18	118.4 (4)	C26—C27—H27	119.7
N2—C8—C13	120.8 (3)	N3—C16—H16A	109.5
N2—C8—C9	118.5 (4)	N3—C16—H16B	109.5
C13—C8—C9	120.7 (4)	N3—C16—H16C	109.5
C23—C18—C17	119.2 (4)	H16A—C16—H16B	109.5
C23—C18—C19	118.0 (4)	H16A—C16—H16C	109.5
C19—C18—C17	122.8 (4)	H16B—C16—H16C	109.5
C13—C12—H12	119.7	N6—C32—H32A	109.5
C11—C12—C13	120.6 (4)	N6—C32—H32B	109.5
C11—C12—H12	119.7	N6—C32—H32C	109.5
C2—C3—H3	119.8	H32A—C32—H32B	109.5
C4—C3—C2	120.4 (4)	H32A—C32—H32C	109.5
C4—C3—H3	119.8	H32B—C32—H32C	109.5
N5—C23—C18	120.6 (4)	N6—C30—O2	122.7 (8)
N5—C23—C22	118.5 (4)	N6—C30—H30	118.6
C18—C23—C22	120.9 (4)	O2—C30—H30	118.6
C12—C11—H11	119.7	N3—C15—H15A	109.5
C12—C11—C10	120.5 (4)	N3—C15—H15B	109.5
C10—C11—H11	119.7	N3—C15—H15C	109.5
C7—C6—H6	120.1	H15A—C15—H15B	109.5
C5—C6—C7	119.8 (4)	H15A—C15—H15C	109.5
C5—C6—H6	120.1	H15B—C15—H15C	109.5
N5—C24—C29	120.0 (4)	N6—C31—H31A	109.5
N5—C24—C25	119.7 (4)	N6—C31—H31B	109.5
C29—C24—C25	120.3 (4)	N6—C31—H31C	109.5
C18—C19—H19	119.8	H31A—C31—H31B	109.5
C20—C19—C18	120.4 (4)	H31A—C31—H31C	109.5
C20—C19—H19	119.8	H31B—C31—H31C	109.5
			
N2—C7—C6—C5	−178.5 (4)	C8—N2—C7—C2	−1.9 (6)
N2—C8—C9—C10	179.3 (4)	C8—N2—C7—C6	177.6 (4)
N4—C17—C18—C23	179.8 (4)	C8—C13—C12—C11	−0.1 (6)
N4—C17—C18—C19	0.5 (7)	C8—C9—C10—C11	0.0 (7)
N1—C1—C13—C8	174.2 (4)	C18—C23—C22—C21	0.1 (7)
N1—C1—C13—C12	−4.1 (6)	C18—C19—C20—C21	−0.3 (7)
N5—C23—C22—C21	179.6 (4)	C12—C13—C8—N2	−179.3 (4)
N5—C24—C25—C26	178.7 (4)	C12—C13—C8—C9	0.9 (6)
C2—C7—C6—C5	1.0 (7)	C12—C11—C10—C9	0.8 (8)
C2—C1—C13—C8	−6.0 (6)	C3—C2—C7—N2	178.7 (4)
C2—C1—C13—C12	175.7 (4)	C3—C2—C7—C6	−0.8 (6)
C2—C3—C4—C5	0.5 (7)	C3—C2—C1—N1	4.9 (6)
C7—N2—C8—C13	1.7 (6)	C3—C2—C1—C13	−174.9 (4)
C7—N2—C8—C9	−178.5 (4)	C3—C4—C5—C6	−0.3 (7)
C7—C2—C1—N1	−174.4 (4)	C23—N5—C24—C29	0.2 (6)
C7—C2—C1—C13	5.8 (6)	C23—N5—C24—C25	−178.9 (4)
C7—C2—C3—C4	0.0 (6)	C23—C18—C19—C20	0.4 (7)
C7—C6—C5—C4	−0.4 (7)	C23—C22—C21—C20	0.0 (8)
C29—C17—C18—C23	−0.1 (6)	C24—N5—C23—C18	−0.9 (7)
C29—C17—C18—C19	−179.4 (4)	C24—N5—C23—C22	179.6 (4)
C29—C24—C25—C26	−0.3 (7)	C24—C29—C17—N4	179.5 (4)
C29—C28—C27—C26	−0.9 (8)	C24—C29—C17—C18	−0.6 (6)
C1—C2—C7—N2	−1.9 (6)	C24—C29—C28—C27	1.0 (7)
C1—C2—C7—C6	178.6 (4)	C24—C25—C26—C27	0.4 (8)
C1—C2—C3—C4	−179.3 (4)	C19—C18—C23—N5	−179.8 (4)
C1—C13—C8—N2	2.4 (6)	C19—C18—C23—C22	−0.3 (7)
C1—C13—C8—C9	−177.5 (4)	C28—C29—C17—N4	−0.6 (7)
C1—C13—C12—C11	178.2 (4)	C28—C29—C17—C18	179.4 (4)
C13—C8—C9—C10	−0.8 (7)	C28—C29—C24—N5	−179.4 (4)
C13—C12—C11—C10	−0.8 (7)	C28—C29—C24—C25	−0.3 (6)
C17—C29—C24—N5	0.6 (6)	C22—C21—C20—C19	0.1 (8)
C17—C29—C24—C25	179.6 (4)	C25—C26—C27—C28	0.2 (8)
C17—C29—C28—C27	−179.0 (5)	C16—N3—C14—O1	−177.2 (6)
C17—C18—C23—N5	0.9 (6)	C32—N6—C30—O2	177.7 (6)
C17—C18—C23—C22	−179.7 (4)	C15—N3—C14—O1	−0.8 (9)
C17—C18—C19—C20	179.7 (4)	C31—N6—C30—O2	−0.1 (10)

### Hydrogen-bond geometry (Å, º)

**Table:**

*D*—H···*A*	*D*—H	H···*A*	*D*···*A*	*D*—H···*A*
N2—H2···O1	0.86	1.86	2.723 (5)	176
N4—H4*A*···Cl2	0.86	2.40	3.225 (3)	160
N4—H4*B*···Cl1	0.86	2.38	3.211 (4)	163
N1—H1*A*···Cl1	0.86	2.39	3.209 (3)	160
N1—H1*B*···Cl2	0.86	2.42	3.246 (3)	162
N5—H5*A*···O2	0.86	1.88	2.740 (5)	175
